# Supplementary material for: Non-Abelian statistics for bosonic symmetry-protected topological phases
Source: Natl Sci Rev. 2026 Jul 21;13(18):nwag444. doi: 10.1093/nsr/nwag444 (PMC13591645; doi:10.1093/nsr/nwag444)
Supplement: Supplementary file 1 [file nwag444_supplemental_file.pdf]

SUPPLEMENTARY MATERIALS

National Science Review
XX: SM, Year
doi: https://doi.org/10.1093/nsr/XXXX

PHYSICS

Non-Abelian statistics for bosonic symmetry-protected topological phases

Hong-Yu Wang[1,2,3,†], Bao-Zong Wang[1,2,3,†], Jian-Song Hong[1,2,3] and Xiong-Jun Liu[1,2,3,*]

[1]International Center for Quantum Materials and School of Physics, Peking University, Beijing 100871, China; [2]International Quantum Academy, Shenzhen 518048, China; [3]Hefei National Laboratory, Hefei 230088, China

*Corresponding authors. E-mails: xiongjunliu@pku.edu.cn. †Equally contributed to this work..

Received: XX XX Year; Revised: XX XX Year; Accepted: XX XX Year

THE FLOQUET THEORY FOR EFFECTIVE HAMILTONIAN

In this section, we derive the effective Hamiltonian Eq. (11) from the periodically driven Hamiltonian

$$H(t) = H_0 + 2H_1' \cos\omega t + 2H_2' \sin\omega t. \quad \text{(S1)}$$

The effective Hamiltonian obtained by Floquet theory up to the order of $1/\omega^2$ is

$$\begin{aligned} H_E &= H_0 + \frac{1}{2\omega^2}([[V_1, H_0], V_{-1}] + \text{ h.c.}) \\ &+\frac{1}{\omega}[V_1, V_{-1}] + O\left(\frac{1}{\omega^3}\right) \\ &= H_0 + \frac{i}{\omega}\left[H_1', H_2'\right] \\ &+\frac{1}{\omega^2}\left[2\left(H_1'H_0H_1' + H_2'H_0H_2'\right)\right. \\ &+\left.\left((H_1')^2H_0 + (H_2')^2H_0 + \text{ h.c.}\right)\right] \\ &+O\left(\frac{1}{\omega^3}\right), \end{aligned} \quad \text{(S2)}$$

where $V_1 = H_1' - iH_2'$ and $V_{-1} = V_1^\dagger$. Substituting

$$\begin{aligned} H_1' &= \delta_1(b_{1,a}^\dagger b_{1,b} + b_{1,b}^\dagger b_{1,a}), \\ H_2' &= \delta_2(ib_{1,a}^\dagger b_{1,b} - ib_{1,b}^\dagger b_{1,a}), \end{aligned} \quad \text{(S3)}$$

into Eq. (S2) gives rise to the Eq. (11).

To identify the zero modes and characterize the effect of Floquet-induced dynamical symmetry breaking, we analyze the low-energy spectrum of the effective Hamiltonian Eq. (11). The parameter $\mu \equiv \frac{\delta_1\delta_2}{\omega}$ characterizes the strength of the dynamical symmetry-breaking term and determines the splitting of the zero-mode degeneracy. Figure S1 shows the ratio between the zero-mode splitting ΔE and the bulk excitation gap E_{gap} as a function of μ. At $\mu = 0$, the two lowest-energy states are separated only by an exponentially small finite-size splitting ΔE, indicating the existence of topological zero modes protected by a finite bulk gap E_{gap}. For weak dynamical symmetry breaking, the condition $\Delta E/E_{\text{gap}} \ll 1$ remains satisfied, and the degenerate ground states remain well separated from the bulk states. As μ increases, ΔE grows and eventually becomes comparable to E_{gap}, indicating the destruction of the zero-mode degeneracy. The condition $\Delta E \ll E_{\text{gap}}$ provides a spectral criterion for identifying the zero modes.

More precisely, whether the boundary modes with finite splitting can still be regarded as zero modes is determined by the condition

$$\Delta E \ll \frac{1}{T} \ll E_{\text{gap}}, \quad \text{(S4)}$$

where T denotes the braiding time. The first inequality ensures that the braiding evolution cannot resolve the zero-mode splitting, while the second corresponds to the adiabatic condition. Once ΔE becomes comparable to E_{gap}, this hierarchy can no longer be satisfied and the fidelity of the zero-mode braiding dynamics is strongly suppressed. Therefore, the ratio $\Delta E/E_{\text{gap}}$ provides a direct spectral diagnostic of the stability of the zero-mode degeneracy and its ability to support non-Abelian braiding statistics.

BRAIDING OPERATORS FROM THE YANG-BAXTER EQUATION

In this section, we derive the braiding operators by solving the Yang-Baxter equation, thereby establishing the SPNA statistics for hard-core bosonic zero modes on a mathematically rigorous footing. We focus on the case protected by the unitary symmetries of particle-hole transformation and particle-number conservation. For

© The Author(s) Year. Published by Oxford University Press on behalf of China Science Publishing & Media Ltd. This is an Open Access article distributed under the terms of the Creative Commons Attribution License (http://creativecommons.org/licenses/by/4.0/), which permits unrestricted reuse, distribution, and reproduction in any medium, provided the original work is properly cited.

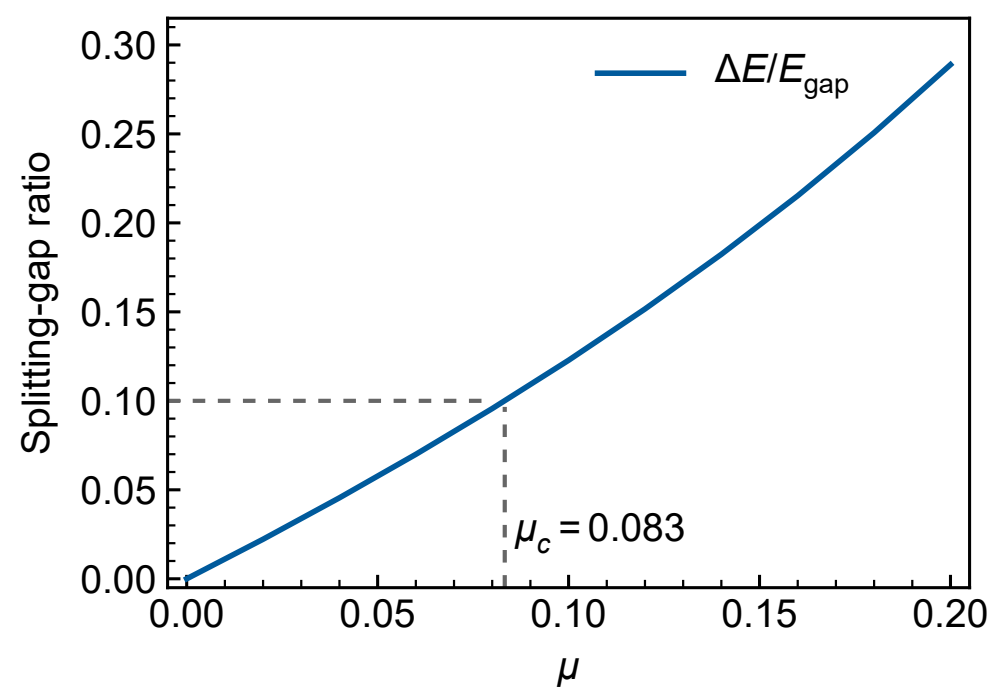

Supplementary Data Figure S1. Ratio between the zero-mode splitting ΔE and the bulk excitation gap $E_{\rm gap}$ as a function of the dynamical symmetry-breaking parameter $\mu = \delta_1\delta_2/\omega$, obtained from exact diagonalization of the effective Hamiltonian Eq. (11) for an open SSH spin chain with $N = 7$ unit cells (14 sites) in the half-filled sector and parameters $v = 1$ and $v' = 0.1$. As μ increases, the Floquet-induced dynamical symmetry-breaking term progressively lifts the zero-mode degeneracy, leading to an increase of $\Delta E/E_{\rm gap}$. The dashed lines mark the characteristic value $\mu_c \simeq 0.083$, defined by $\Delta E/E_{\rm gap} = 0.1$. For $\mu < \mu_c$, the condition $\Delta E \ll E_{\rm gap}$ remains well satisfied and the degenerate ground states remain clearly separated from bulk excitations. Beyond this regime, the zero-mode splitting becomes increasingly comparable to the bulk gap.

two zero modes, the most general effective Hamiltonian governing the braiding process is:

$$H_E = \lambda G_{12}(\alpha) + \delta P_{12}, \tag{S5}$$

where $\lambda, \delta \in \mathbb{R}$, $G_{12}(\alpha) \equiv e^{i\alpha}\beta_1^\dagger\beta_2 + e^{-i\alpha}\beta_2^\dagger\beta_1$ with $\alpha \in [0, 2\pi)$, and $P_{12} \equiv G_{12}^2 = n_1 + n_2 - 2n_1n_2$ is the projector onto the half-filled subspace $\mathcal{H}_0$. These operators satisfy

$$G^3 = GP = PG = G, \quad P^2 = P. \tag{S6}$$

The braiding operator takes the form

$$\begin{aligned} U_{12} &= e^{-iH_ET} = e^{-i\lambda T G_{12}(\alpha)}e^{-i\delta T P_{12}} \\ &= I - ie^{-i\theta}\sin(\varphi)G_{12}(\alpha) \\ &+ (e^{-i\theta}\cos(\varphi) - 1)P_{12}, \end{aligned} \tag{S7}$$

where the subscript indicates the zero-mode pair being exchanged, and we define $\lambda T \equiv \varphi$ and $\delta T \equiv \theta$ for brevity.

When more than two zero modes are present, the Yang-Baxter equation [1] ensures consistency between two different sequences of braiding three zero modes:

$$U_{12}U_{23}U_{12} = U_{23}U_{12}U_{23}. \tag{S8}$$

substituting Eq. (S7) into Eq. (S8), we obtain the equation

$$\begin{aligned} (a + 2ab &+ ab^2)(G_{12}(\alpha) - G_{23}(\alpha)) \\ &+ (a^2 + b + b^2 + a^2b)(P_{12} - P_{23}) \\ &= 0, \end{aligned} \tag{S9}$$

where we have defined $a \equiv -ie^{-i\theta}\sin(\varphi)$ and $b \equiv e^{-i\theta}\cos(\varphi) - 1$, and used the algebraic relations

$$[P_{12}, P_{23}] = 0 \tag{S10}$$
$$\{P_{12}, G_{23}(\alpha)\} = G_{23}(\alpha), \tag{S11}$$
$$\{P_{23}, G_{12}(\alpha)\} = G_{12}(\alpha), \tag{S12}$$

to simplify intermediate steps. Thus, a and b must satisfy

$$a+2ab+ab^2 = 0, \quad a^2+b+b^2+a^2b = 0. \tag{S13}$$

For simplicity and without loss of physical content, we restrict θ and φ to the range $(-\pi, \pi]$; more general solutions are $\theta + 2n\pi$ and $\varphi + 2m\pi$ with $m, n \in \mathbb{Z}$. The nontrivial solutions of Eq. (S13) are $\varphi = \pm\frac{\pi}{2}$. The choice of the plus or minus sign is merely a convention, as the two cases are related by Hermitian conjugation accompanied by reversing the sign of θ. Physically, the sign corresponds to the braiding direction—clockwise or counterclockwise. In this work, we adopt the $\varphi = -\frac{\pi}{2}$ convention, and the corresponding braiding operator is

$$U_{12} = I + ie^{-i\theta}G_{12}(\alpha) - P_{12}. \tag{S14}$$

The solution of the braiding operator in Eq. (S14) to the Yang-Baxter equation depends on a continuous parameter θ. This dependence originates from the term $\propto P_{12}$ in H_E, which physically describes an interaction between the two zero modes: $P_{12} = 1$ when exactly one zero mode is occupied and $P_{12} = 0$ otherwise. Such an interaction leads to phase accumulation during braiding, thereby producing a continuous family of solutions for the braiding operator. The fact that the Hamiltonian is real imposes an additional constraint: the Berry phase must be real. Consequently, for a full braiding process, the corresponding transformation is

$$U_{12}^2 = 1 - (1 + e^{-2i\theta})P_{12} = \pm I. \tag{S15}$$

We refer to the cases $U^2 = I$ and $U^2 = -I$ as conventional braiding statistics and defect-assisted braiding statistics, respectively. When both zero modes are either occupied or empty, $P_{12} = 0$, yielding $U_{12}^2 = 1$. This result is physically consistent because the bosonic commutation relations of the zero-mode operators require that the singlet sectors $|0,0\rangle$ and $|1,1\rangle$ are invariant under braiding. Nevertheless, when exactly one zero mode is occupied, we have $U_{12}^2 = -e^{-2i\theta}$. This condition leads to two distinct classes of solutions for the parameter θ: (i) $\theta = \pm\pi/2$, and (ii) $\theta = 0$ or π, corresponding to two classes of effective Hamiltonians. Moreover, as shown in Fig. 3

and in the Appendix, the braiding transformation of the ground states is real. This requires that the coefficients in Eq. (S14) be real, which in turn fixes α with a given θ. For the first class, the effective Hamiltonian realizes the conventional braiding statistics, with the braiding operator

$$\begin{aligned} U_{12}^{\text{conv}} &= I + G_{12}(0) - P_{12}, \\ &= I + \beta_1^\dagger\beta_2 + \beta_2^\dagger\beta_1 - n_1 - n_2 + 2n_1n_2, \end{aligned} \tag{S16}$$

where we take $\theta = \pi/2$ by convention, as the opposite choice only introduces an overall -1 factor to the braiding matrix in the subspace $\mathcal{H}_e$. Since the coefficients in Eq. (S16) are already real, the α takes the value 0 (Also, $\alpha = \pi$ only contributes an overall factor of -1).

For the second class, we choose $\theta = 0$ with the associated $\alpha = \frac{\pi}{2}$ to ensure that the braiding operator is real. It then reads

$$\begin{aligned} U_{12}^{\text{def}} &= I + iG_{12}(\frac{\pi}{2}) - P_{12}, \\ &= I - \beta_1^\dagger\beta_2 + \beta_2^\dagger\beta_1 - n_1 - n_2 + 2n_1n_2, \end{aligned} \tag{S17}$$

where the opposite choice differs only by Hermitian conjugation. These results, Eqs. (S16) and (S17), are confirmed with the numerical results and the exact solution of the wavefunction evolution during the braiding process, in which all the coefficients are real, as shown in the following appendix section.

THE NON-ABELIAN BERRY PHASE AND TOPOLOGICAL CLASSIFICATION

In this section, we compute the non-Abelian Berry phase associated with the braiding process using the Wilczek-Zee connection. We show that conventional and defect-assisted braiding statistics belong to distinct topological classes and provide a geometric interpretation for both classes of non-Abelian statistics.

The Wilczek-Zee connection generalizes Berry's geometric phase to the case of degenerate states [2]. Consider a Hamiltonian $H(\vec{R})$ with a degenerate subspace of energy E_m, spanned by orthonormal basis $\{|\psi_{m,a}\rangle\}$ with $a = 1, ..., d_m$ denoting the multiplicity, and depending on time through parameters $\vec{R}(t)$. Under a time-dependent evolution $\vec{R}(t)$, the state evolves as

$$|\Psi(t)\rangle = \sum_{m,a} e^{-i\int E_m(t)dt} c_{m,a}(t) \left|\psi_{m,a}(t)\right\rangle, \tag{S18}$$

where $|\psi_{m,a}(t)\rangle$ are instantaneous orthonormal eigenstates $H(\vec{R}(t))\left|\psi_{m,a}(t)\right\rangle = E_m(t)\left|\psi_{m,a}(t)\right\rangle$. The Schrödinger equation then gives

$$\begin{aligned} &\dot{c}_{n,b}(t) \\ &= -\sum_a \langle\psi_{n,b}(t)|\dot{\psi}_{n,a}(t)\rangle c_{n,a}(t) \\ &- \sum_{m\neq n,a} c_{m,a}(t)e^{-i\int(E_m-E_n)dt} \\ &\quad \times\frac{\langle\psi_{n,b}(t)|\dot{H}(t)|\psi_{m,a}\rangle}{E_m-E_n} \\ &\approx -\sum_a \langle\psi_{n,b}(t)|\dot{\psi}_{n,a}(t)\rangle c_{n,a}(t) \\ &=: -\sum_a A_{ba}^n(t)c_{n,a}(t), \end{aligned} \tag{S19}$$

where we use the adiabatic approximation

$$|\frac{\langle\psi_{n,b}(t)|\dot{H}(t)|\psi_{m,a}\rangle}{E_m-E_n}| \ll |A_{ba}^n|.$$

Here, $A_{ba}^n \equiv \langle\psi_{n,b}(t)|\dot{\psi}_{n,a}(t)\rangle$ is the Wilczek-Zee connection. The formal solution of Eq. (S19) is

$$c_{n,b}(t) = \sum_a \mathcal{P}e^{-\int A_{ba}^n(t)dt}c_{n,a}(0), \tag{S20}$$

where $\mathcal{P}$ denotes path ordering.

For simplicity, we focus on the minimal braiding setups illustrated in Fig. 5(b) and Fig. 5(c). The extension to larger tri-junction configurations is straightforward. The Hamiltonian of the minimal setup is

$$H(\vec{R}(t)) = v_1(t)b_1^\dagger b_2 + v_2(t)b_2^\dagger b_3 + v_3(t)b_2^\dagger b_4 + h.c., \tag{S21}$$

where the parameter vector is defined as $\vec{R} = (v_1, v_2, v_3)^T$, with $v_1(t)$, $v_2(t)$, and $v_3(t)$ time-dependent coupling parameters, e.g., illustrated in Fig. 5(d). The parameter space consists of three patches joined along their edges, forming a conical surface with a singular point at $\vec{R} = (0,0,0)^T$ where the gap closes. The braiding process corresponds to a closed loop encircling this singularity, which gives rise to a non-Abelian Berry phase. Because the Hamiltonian is real, one can always choose a real instantaneous basis at each time through an appropriate gauge choice. Consequently, the Wilczek-Zee connection is purely real and satisfies

$$A_{aa}^n = 0, \qquad A_{ab}^n = -A_{ba}^n. \tag{S22}$$

We first consider defect-assisted braiding. In this case, the system is occupied by a single

hard-core boson, and at $t = 0$ the zero-energy subspace (the ground-state subspace in the presence of a local defect) is spanned by the basis states $(|1\rangle, |4\rangle)$, where $|j\rangle \equiv b_j^\dagger |\Uparrow\rangle$ with $|\Uparrow\rangle$ denoting the state in which all spins are in the up state.

To ensure smooth evolution of the basis vectors, we adopt the following gauge choice:

$$\begin{cases} \left(\frac{v(t)|1\rangle - u(t)|3\rangle}{\sqrt{v(t)^2+u(t)^2}}, |4\rangle\right), & 0 \le t < T_0 \\ \left(-|3\rangle, \frac{v(t-T_0)|4\rangle - u(t-T_0)|1\rangle}{\sqrt{v(t-T_0)^2+u(t-T_0)^2}}\right), & T_0 \le t < 2T_0 \\ \left(\frac{-v(t-2T_0)|3\rangle + u(t-2T_0)|4\rangle}{\sqrt{v(t-2T_0)^2+u(t-2T_0)^2}}, -|1\rangle\right), & 2T_0 \le t \le 3T_0 \end{cases} \tag{S23}$$

where $u(t)$ and $v(t)$ are smooth interpolation functions satisfying the boundary conditions $u(0) = 0$, $u(T_0) = 1$, $v(0) = 1$ and $v(T_0) = 0$.

For conventional braiding, the system is occupied by two hard-core bosons. At $t = 0$, the ground-state subspace is spanned by the basis $\left(\frac{1}{\sqrt{2}}(|12\rangle - |13\rangle), \frac{1}{\sqrt{2}}(|24\rangle - |34\rangle)\right)$, where $|ij\rangle = b_i^\dagger b_j^\dagger |\Uparrow\rangle$. With an appropriate gauge choice, the basis states evolve smoothly as

$$\begin{cases} \left(\frac{v(t)\,|12\rangle + u(t)\,|23\rangle - \sqrt{u(t)^2+v(t)^2}\,|13\rangle}{\sqrt{2(u(t)^2+v(t)^2)}}, \frac{-v(t)\,|34\rangle - u(t)\,|14\rangle + \sqrt{u(t)^2+v(t)^2}\,|24\rangle}{\sqrt{2(u(t)^2+v(t)^2)}}\right), & 0 \le t < T_0, \\ \left(\frac{-v(t')\,|13\rangle - u(t')\,|34\rangle + \sqrt{u(t')^2+v(t')^2}\,|23\rangle}{\sqrt{2(u(t')^2+v(t')^2)}}, \frac{v(t')\,|24\rangle + u(t')\,|12\rangle - \sqrt{u(t')^2+v(t')^2}\,|14\rangle}{\sqrt{2(u(t')^2+v(t')^2)}}\right), & 0 \le t' < T_0, \\ \left(\frac{v(t'')\,|23\rangle + u(t'')\,|24\rangle - \sqrt{u(t'')^2+v(t'')^2}\,|34\rangle}{\sqrt{2(u(t'')^2+v(t'')^2)}}, \frac{-v(t'')\,|14\rangle - u(t'')\,|13\rangle + \sqrt{u(t'')^2+v(t'')^2}\,|12\rangle}{\sqrt{2(u(t'')^2+v(t'')^2)}}\right), & 0 \le t'' \le T_0. \end{cases} \tag{S24}$$

where $t' \equiv t - T_0$ and $t'' \equiv t - 2T_0$. Eqs. (S23) and (S24) show that the basis states are changed at the end of the braiding. To compute the non-Abelian Berry phase, we adiabatically return the basis to its initial configuration using the following gauge transformations for the time interval $2T_0 \le t < 3T_0$:

$$\text{defect-assisted}: \quad g^{\text{def}} = e^{i\phi(t)\sigma^y}, \tag{S25}$$

$$\text{conventional}: \quad g^{\text{conv}} = \sigma^z e^{i\phi(t)\sigma^y}. \tag{S26}$$

Here, $\phi(t)$ is a smooth function satisfying $\phi(2T_0) = 0$ and $\phi(3T_0) = \frac{\pi}{2}$. These gauge transformations act on the basis from the right, i.e., $(|\psi_1\rangle, |\psi_2\rangle) \cdot g$. The Wilczek-Zee connection is then given by

$$A = i\sigma^y \dot{\phi}(t), \tag{S27}$$

for both defect-assisted braiding and conventional braiding. The corresponding non-Abelian Berry phase is

$$U^{\text{def}} = \mathcal{P}e^{-\int_{2T_0}^{3T_0} A dt} = -i\sigma^y \in SO(2), \tag{S28}$$

$$U^{\text{conv}} = \mathcal{P}e^{-\int_{2T_0}^{3T_0} A dt}\sigma^z = \sigma^x \in O(2). \tag{S29}$$

The key difference between U^{def} and U^{conv} is the additional reflection operation σ^z in the conventional braiding. This operation acts on the zero-mode subspace as a matrix-valued transformation rather than an overall $U(1)$ phase factor, and is responsible for the non-commutativity between the two classes of braiding statistics. Therefore, the distinction does not originate from different types of zero modes, but from different topological sectors of the braiding process. The insertion of a local defect introduces a branch cut in the braiding parameter space, so braiding paths involving the same zero modes but different defect configurations can belong to distinct topological sectors. Crossing this branch cut changes the braiding transformation by an additional σ^z operation, leading to the two distinct classes of braiding statistics derived below. In conventional braiding, this reflection acts as a branch cut: crossing it flips the orientation of the basis vectors, so the subsequent phase accumulation changes sign.

Mathematically, the determinants of two braiding matrices $\det(U^{\text{def}}) = 1$ and $\det(U^{\text{conv}}) = -1$, arising from the fact that a real system only allows phase factors ± 1, correspond to two topological invariants associated with the first Stiefel-Whitney class of a real vector bundle. More precisely, the evolution of the two-dimensional zero-mode subspace defines a rank-2 real vector bundle E over the base manifold S^1, which corresponds to a closed loop

in parameter space. Since the basis states are real and normalized, each fiber is a circle S^1, so E is a circle bundle over a circle. Such bundles fall into two distinct classes: the orientable torus and the non-orientable Klein bottle. Their classification is given by the first Stiefel-Whitney class, defined as

$$w_1(E) := \frac{1-\det(U)}{2} \in H^1(S^1;\mathbb{Z}_2) \cong \mathbb{Z}_2. \tag{S30}$$

The class $w_1(E) = 0$ corresponds to an orientable bundle, where the structure group reduces from $O(2)$ to $SO(2)$, matching the defect-assisted braiding. The class $w_1(E) = 1$ corresponds to a non-orientable bundle, where the structure group remains $O(2)$, as in the conventional braiding. We summarize the correspondences in Table. S1. This provides a topological interpretation of the two classes of braiding statistics.

AVERAGE FIDELITY AND UNITARITY

We introduce average fidelity as a quantitative measure to evaluate the discrepancy between actual and ideal braiding results [3]. Given an arbitrary initial state $|\psi\rangle$, the fidelity of an operator U can be characterized by

$$f(U,O) = |\langle\psi|O^\dagger U|\psi\rangle|^2, \tag{S31}$$

where $O|\psi\rangle$ defines the final state after an ideal operation. Then the average fidelity is defined as the fidelity averaged over the subspace encoding the qubit: $F(U,O) \equiv \int_{S^3} |\langle\psi|O^\dagger U|\psi\rangle|$, which yields

$$F(U,O) = \frac{1}{6}(\mathrm{Tr}(U^\dagger U) + |\mathrm{Tr}(O^\dagger U)|^2). \tag{S32}$$

This quantity characterizes the agreement between the outcomes of the operator U and the ideal operator O when acting on states within the qubit subspace. For our study, the ideal braiding matrix O is either $i\sigma_y$ for the defect-assisted unconventional braiding or σ^z for the conventional braiding in the basis $\{|\eta\rangle, |\xi\rangle\}$. If the operator U is unitary, the average fidelity has a lower bound

$$F_{\min}(U,O) = \frac{1}{3}. \tag{S33}$$

To quantify how unitary the braiding matrix U is, we further define the following quantity:

$$\begin{aligned}\tilde{U}(U) &\equiv \int_{S^3} |\langle\psi|U^\dagger U|\psi\rangle| \\ &= \frac{1}{6}(\mathrm{Tr}(U^\dagger U U^\dagger U) + |\mathrm{Tr}(U^\dagger U)|^2).\end{aligned} \tag{S34}$$

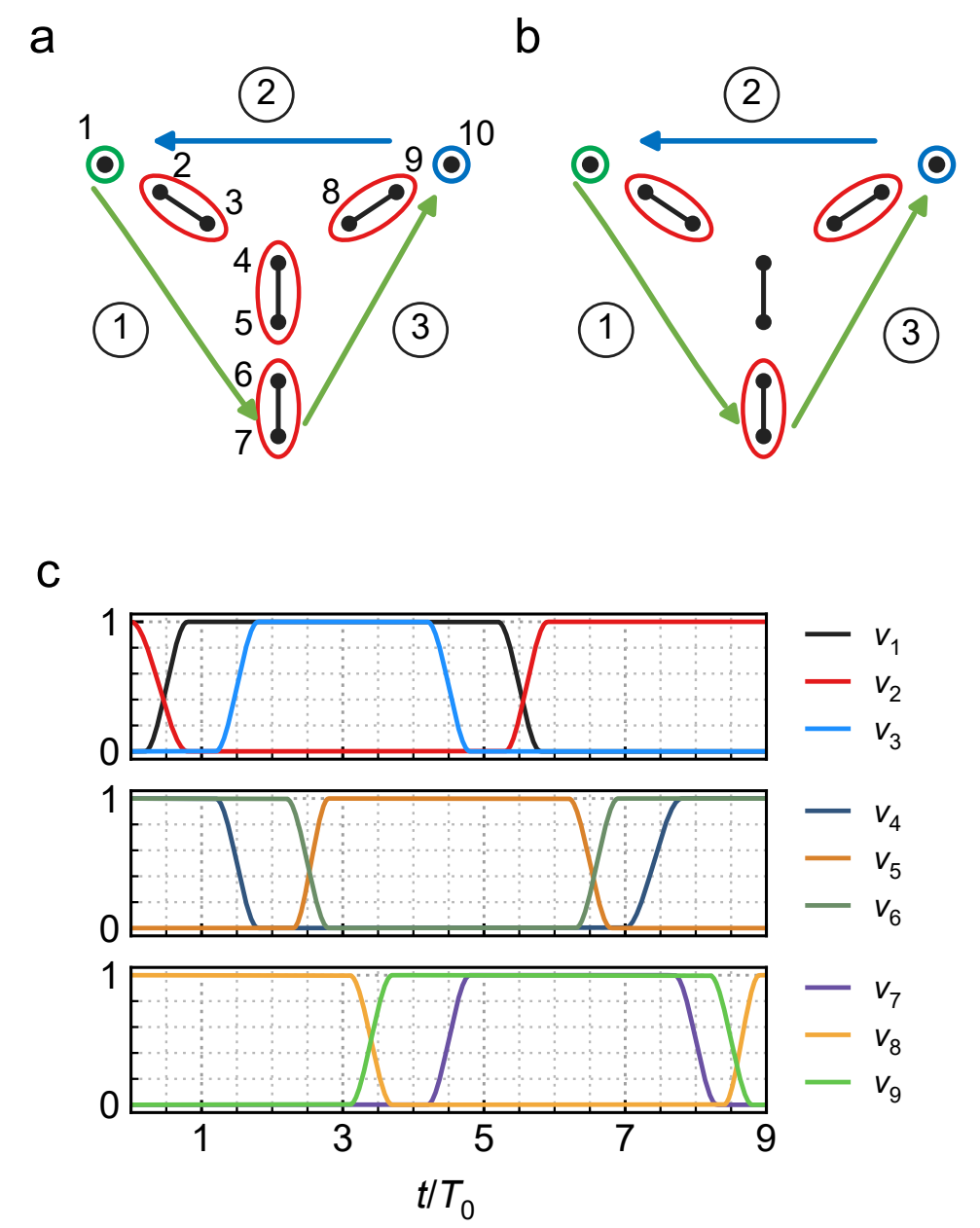

Supplementary Data Figure S2. Tri-junctions in the dimerized limit with 10 sites, where site 1 (10) hosts the left (right) zero mode. The ground state $|\beta_1\rangle$ ($|\beta_2\rangle$) corresponds to site 1 (10) being occupied. The green and blue arrows indicate the direction in which the zero mode moves during braiding. (a) The tri-junction that realizes the conventional braiding. (b) The tri-junction that realizes the defect-assisted braiding, with sites 4 and 5 serving as the local defect. (c) Time dependence of coupling strengths in the dimerized tri-junction for braiding realization. The horizontal axis denotes the number of elementary steps.

The braiding matrix U is unitary if and only if $\tilde{U}(U) = 1$.

CALCULATIONS IN THE DIMERIZED LIMIT

In this section, we study the two classes of braiding statistics in the dimerized limit, which provides a more intuitive understanding of the non-trivial geometric phases. We focus on specific dimerized tri-junctions that realize the first [Fig. S2(a)] and the second [Fig. S2(b)] types of braiding scheme.

We first compute the geometric phase of an elementary moving step illustrated in Fig. 1(e). The dynamics of an elementary moving step is governed by the Hamiltonian H_e in Eq. (24). In the dimerized limit, the parameters at $t = 0$ are initialized with $u(0) = 0$, $v(0) = v$. Adiabatically tuning $u(t)$ to $u(T_0) = v$ and $v(t)$ to $v(T_0) = 0$ moves the zero mode from site 1 to site 3. The geometric phase obtained by the zero mode depends on the particle-number occupation on sites 2 and 3. In both the single-particle subspace spanned by $\{b_1^\dagger|\Uparrow\rangle, b_2^\dagger|\Uparrow\rangle, b_3^\dagger|\Uparrow\rangle\}$ and the two-particle subspace spanned by $\{b_2^\dagger b_3^\dagger|\Uparrow$

the first Stiefel-Whitney class	topological invariant	braiding matrix	structure group
$w_1(E) = 0$	$\det(U^{\mathrm{def}}) = 1$	$U^{\mathrm{def}} = -i\sigma^y$	SO(2)
$w_1(E) = 1$	$\det(U^{\mathrm{conv}}) = -1$	$U^{\mathrm{conv}} = \sigma^x$	O(2)

Table S1. Correspondences between the first Stiefel-Whitney class, the associated topological invariants, the two classes of braiding statistics, and structure groups.

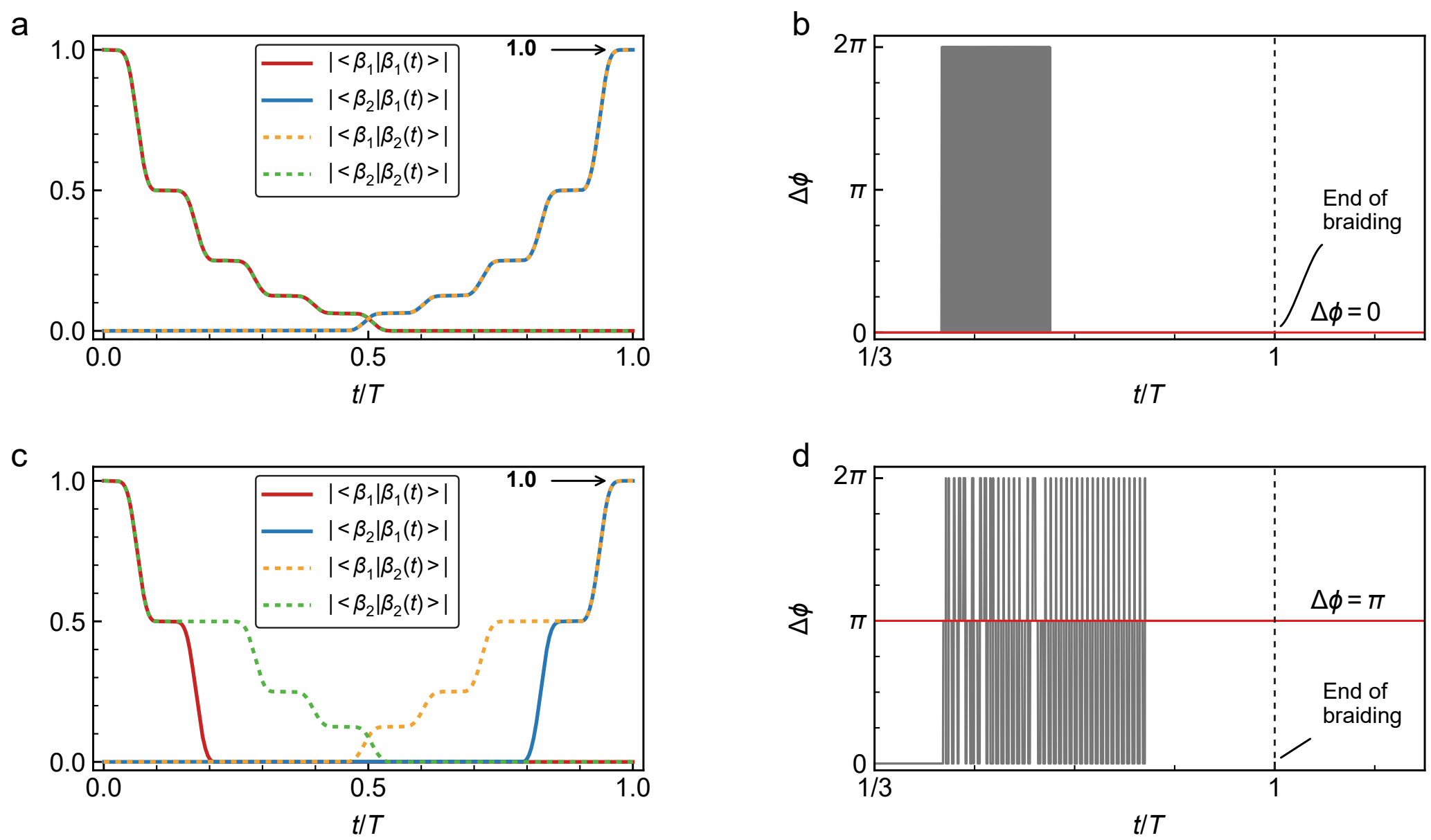

Supplementary Data Figure S3. Numerical time evolution of zero-mode wavefunctions for two braiding schemes in the dimerized limit. (a) Amplitudes of and (b) phase difference between $|\beta_1(t)\rangle$ and $|\beta_2(t)\rangle$ for the first type of braiding scheme (conventional braiding). (c) Amplitudes and (d) phase difference for the second type of braiding scheme (defect-assisted braiding). The horizontal axis represents the evolution time in units of the total braiding time T. The strong and weak couplings take $v = 1$ and $v' = 0$. Simulations are performed with a time step $\Delta t = 0.1$ and an elementary step time $T_0 = 80$. Each braiding involves 9 elementary steps of zero-mode motion, yielding a total braiding time $T = 9T_0 = 720$. The red horizontal line indicates the theoretical prediction of $\Delta\phi = \mathrm{Arg}(\langle\beta_2|\beta_1(t)\rangle) - \mathrm{Arg}(\langle\beta_1|\beta_2(t)\rangle) \bmod 2\pi$ for both schemes. The vertical dashed line marks the completion of the braiding process, after which the system is held static to show the stabilized value of $\Delta\phi$. The numerical results of $\Delta\phi$ for both classes of statistics are indicated in the figure.

$\rangle, b_3^\dagger b_1^\dagger|\Uparrow\rangle, b_1^\dagger b_2^\dagger|\Uparrow\rangle\}$, where $|\Uparrow\rangle$ denotes the state with all spins up, the H_e is

$$H_\mathrm{e} = \begin{pmatrix} 0 & u(t) & 0 \\ u(t) & 0 & v(t) \\ 0 & v(t) & 0 \end{pmatrix}. \tag{S35}$$

The initial states are eigenstates of H_e with site 1 occupied. In the single-particle subspace, the initial state is the zero-energy state $\left[v(t)b_1^\dagger - u(t)b_3^\dagger\right]|\Uparrow\rangle$. In the two-particle subspace, the initial state is the ground state $\left[v(t)b_1^\dagger b_2^\dagger + u(t)b_2^\dagger b_3^\dagger - \sqrt{u(t)^2 + v(t)^2} b_3^\dagger b_1^\dagger\right]|\Uparrow\rangle$. Then after the elementary moving step $u(t) : 0 \to v$, $v(t) : v \to 0$, these states evolve as follows:

$$b_1^\dagger|\Uparrow\rangle \rightarrow -b_3^\dagger|\Uparrow\rangle, \tag{S36}$$

$$b_1^\dagger(b_2^\dagger - b_3^\dagger)|\Uparrow\rangle \rightarrow b_3^\dagger(b_2^\dagger - b_1^\dagger)|\Uparrow\rangle. \tag{S37}$$

In the single-particle subspace, the zero mode changes its position and acquires a π phase. In the two-particle subspace, the zero mode simply changes its position without acquiring an additional phase.

We now consider the specific dimerized tri-junctions depicted in Fig. S2(a) and (b). The braiding process involves 9 elementary moving steps with a total braiding time $T = 9T_0$. In the ground state of the dimerized tri-junction, hard-core bosons form dimer bond states (i.e., Bell states in the spin representation) between pairs of sites. In Fig. S2(a), all sites except for edge sites 1 and 10 are occupied by dimers. In Fig. S2(b), the pair sites $(4, 5)$ are not occupied by a dimer and play the role of a local defect. During the braiding process, the filling on $(4, 5)$ is crucial to the net geometric phase because it is the only pair of sites through which one zero mode passes once while another passes twice.

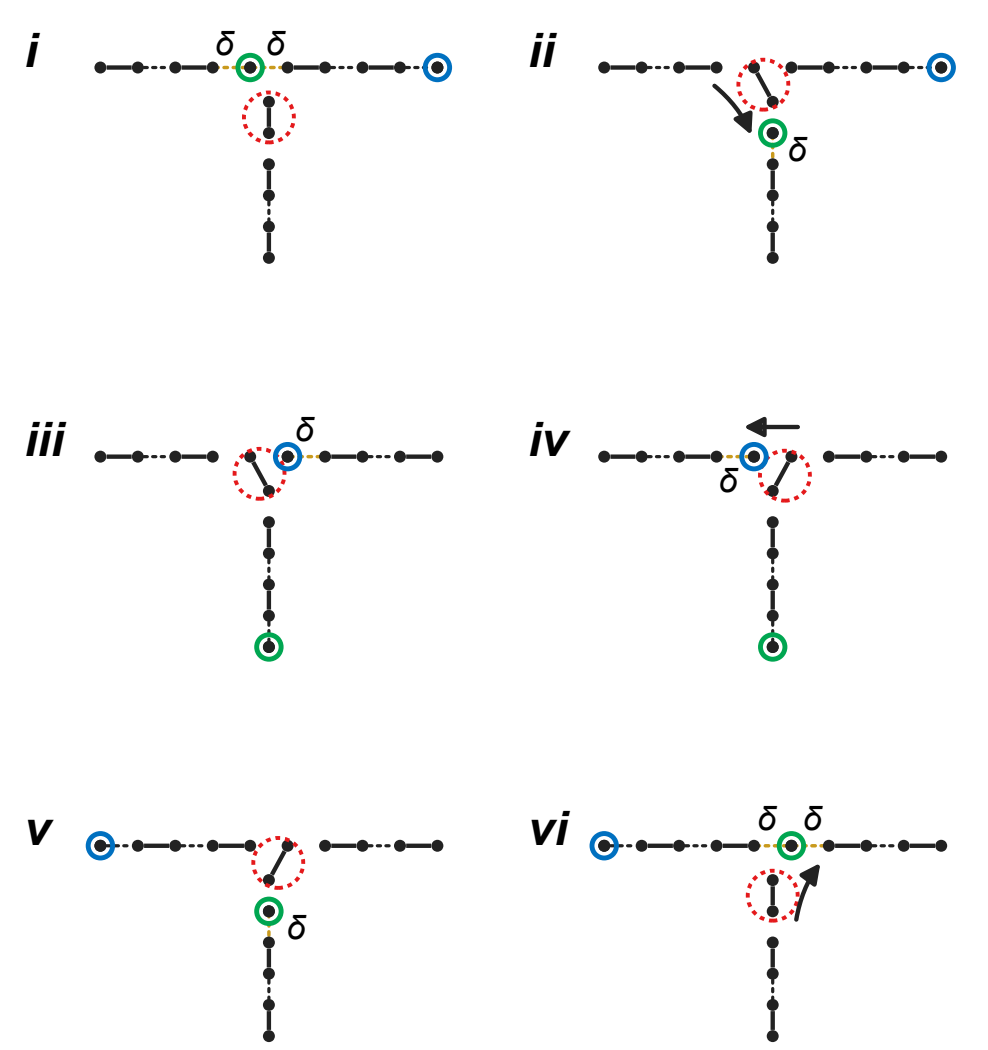

Supplementary Data Figure S4. Imperfect defect-assisted braiding procedure. The golden dotted lines represent the residual coupling strength δ between the zero modes and other parts of the tri-junction as the zero modes pass through the local defect.

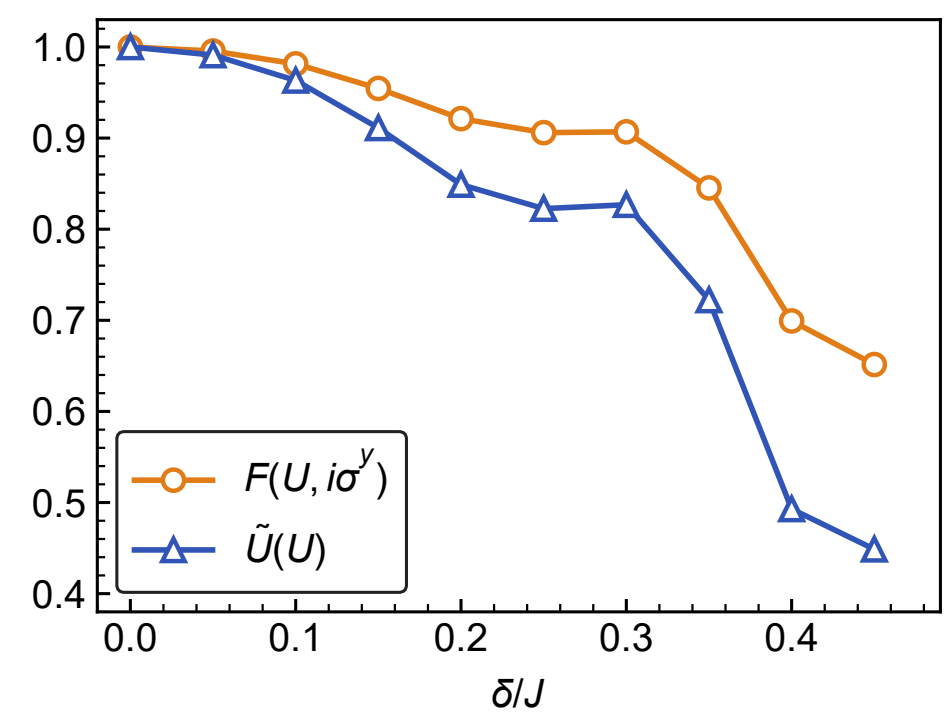

Supplementary Data Figure S5. Numerical calculations of the average fidelity F and unitarity $\tilde{U}$ as functions of the residual coupling strength δ (in units of the maximal coupling strength J) for imperfect defect-assisted braiding procedures.

The other pairs are either passed through once by both zero modes or twice by a single zero mode and thus do not contribute to the net geometric phase. In the case of Fig. S2(a), since the local defect $(4, 5)$ is half-filled, ground states acquire no geometric phase difference. Therefore, states $|\beta_1\rangle$ and $|\beta_2\rangle$ transform as Eq. (27). In the case of Fig. S2(b), however, the local defect $(4, 5)$ is empty, ground states $|\beta_1\rangle$ and $|\beta_2\rangle$ acquire a π geometric phase difference. Accordingly, states $|\beta_1\rangle$ and $|\beta_2\rangle$ transform as Eq. (28).

We numerically verify the above theoretical results. The time-dependent Hamiltonian used to implement both braiding schemes with tri-junctions is

$$H(t) = \sum_{i=1,2,\ldots,6,8,9} v_i(t) b_{i+1}^{\dagger} b_i + v_7(t) b_8^{\dagger} b_4 + h.c. \tag{S38}$$

In the braiding, we adjust the coupling strengths over time following the functions depicted in Fig. S2(c). The rising (falling) slope in Fig. S2(c) follows the form $\chi(t/T_0)$ $(1-\chi(t/T_0))$ defined in Eq. (26). The numerical results, shown in Fig. S3, match the theoretical predictions.

ERROR PREVENTION THROUGH LOCAL DEFECT ISOLATION

Defect-assisted braiding requires isolating the local defect to ensure that it returns to the original state after braiding. Failure to meet this condition causes errors in the braiding results. In the following, we analyze these errors and verify that our scheme of isolating the local defect effectively prevents them. Specifically, we examine an imperfect defect-assisted braiding procedure where the zero mode is not fully localized before and after passing through the local defect, but retains a residual coupling δ with a portion of the tri-junction, as shown in Fig. S4. We numerically compute the change in average fidelity as a function of the residual coupling strength δ. The results, shown in Fig. S5, confirm that our scheme (with $\delta = 0$) effectively prevents errors.

We also calculate the unitarity (defined in Appendix) of the braiding matrix. As shown in Fig. S5, the unitarity follows a behavior similar to that of the average fidelity, confirming that errors arise when the local defect fails to fully return to its original state. This leads to components outside the subspace $\mathcal{H}_0$ in the states $|\beta_1(T)\rangle$ and $|\beta_2(T)\rangle$, resulting in a non-unitary braiding matrix. Setting $\delta = 0$ in perfect defect-assisted braiding solves this problem. It also suggests that measuring the local defect state offers a practical method to assess braiding results.

REFERENCES

1. Drinfeld VG. Hopf algebras and the quantum Yang-Baxter equation. *Sov Math Dokl* 1985; **32**: 254–8.
2. Wilczek F and Zee A. Appearance of gauge structure in simple dynamical systems. *Phys Rev Lett* 1984; **52**: 2111–14.
3. Pedersen LH, Møller NM and Mølmer K. Fidelity of quantum operations. *Phys Lett A* 2007; **367**: 47–51.
